# Bridging the life-course exposome approach with a life-cycle perspective in safe and sustainable by design (SSbD) for chemical risk

**DOI:** 10.1186/s40246-026-00976-1

**Published:** 2026-05-07

**Authors:** Dimosthenis Sarigiannis, Fotini Nikiforou, Achilleas Karakoltzidis, Nafsika Papaioannou, Spyros Karakitsios

**Affiliations:** 1 National Hellenic Research Foundation, 48 Vassileos Constantinou Avenue, 11635 Athens, Greece; 2 Department of Chemical Engineering, Environmental Engineering Laboratory, Aristotle University of Thessaloniki, University Campus, 54124 Thessaloniki, Greece; 3 HERACLES Research Center on the Exposome and Health, Center for Interdisciplinary Research and Innovation, Aristotle University of Thessaloniki, Balkan Center, Bldg. B, 10th km Thessaloniki-Thermi Road, 57001 Thessaloniki, Greece; 4 Science, Technology and Society Department, Environmental Health Engineering, University School for Advanced Study (IUSS), Piazza della Vittoria 15, 27100 Pavia, Italy; 5 Department of Urology, Medical School, Johns Hopkins University, MD 21287 Baltimore, USA; 6 School of Public Health, Yale University, Connecticut 06510 New Haven, USA

**Keywords:** Safe and sustainable by design, Exposome, AI, NAMs

## Abstract

Safe and Sustainable by Design (SSbD) and life‑course exposome science share a primary‑prevention goal: reducing harmful exposures by intervening early in the chemical and product life cycle. Here we propose an integrated, replacement‑first decision workflow that links life‑course exposure considerations to SSbD stage‑gates using New Approach Methodologies (NAMs). The framework combines: (i) problem formulation informed by susceptible windows and populations; (ii) AOP‑guided selection and curation of mechanistic in vitro bioactivity evidence (e.g., ToxCast/Tox21, transcriptomics); (iii) physiologically based pharmacokinetic (PBPK) modelling with quantitative in vitro‑to‑in vivo extrapolation to translate in vitro potency into internal dose anchors; and (iv) transparent multi‑criteria synthesis to support early design trade‑offs. We illustrate this approach with an endocrine‑relevant substitution scenario comparing bisphenol A (BPA) with a structurally similar alternative (BPAP) and a bio‑based monomer candidate (isosorbide). Estrogen receptor (ER) bioactivity anchors derived from curated HTS assays are contrasted with PBPK‑predicted internal concentration ranges to generate an internal margin for stage‑gate decisions. The example shows how candidates with weak or absent pathway‑relevant bioactivity can be advanced, while structurally similar alternatives with ER potency approaching predicted internal concentrations can be deprioritised or redesigned pending improved exposure controls. By explicitly mapping NAM outputs to AOP key events and life‑course windows, the workflow operationalises the 3Rs by replacing broad exploratory animal testing with targeted, human‑relevant evidence and reducing unnecessary in vivo studies through early prioritisation.

## Highlights

- Proposes an integrated life-course exposome–SSbD decision workflow.
- Links AOP-anchored NAM bioactivity to PBPK-derived internal dose metrics.
- Operationalises stage-gate decisions using exposure-led NGRA principles.
- Demonstrates avoidance of regrettable substitution (BPA vs BPAP).
- Identifies isosorbide as a safer, exposome-informed SSbD alternative.

## Introduction

Over the last two decades, thinking about chemical exposure and health has shifted from single substances and discrete time points toward dynamic, systems-based views. One key development is first articulated by Wild [1], the exposome, that captures the totality of environmental exposures across the lifespan, highlighting critical windows of susceptibility.

In parallel, the European Safe and Sustainable by Design (SSbD) initiative has emerged as a forward-looking approach to chemicals and product development. SSbD focuses on identifying and eliminating risks at the design stage [2, 3], instead of relying on protracted downstream debates about substitution. It adopts a full life-cycle perspective, from raw material extraction through manufacturing, use, recycling and end-of-life, with the aim of embedding safety, efficiency and sustainability at each step [4].

SSbD is a prevention-oriented framework that seeks to eliminate chemical risks at the design stage rather than managing them downstream [5], while the exposome underscores that harmful exposures occurring during susceptibility windows [6], over the life course [7].

This paper first examines the conceptual foundations of SSbD and its links to green chemistry, pollution prevention and circular economy principles. It then turns to the life-course exposome, outlining how biomonitoring, mixture toxicity and vulnerable life stages challenge conventional risk assessment. Next, it explores the points of convergence between these two frameworks and proposes practical steps, improving data interoperability, fostering cross-disciplinary collaboration and adopting new decision-support tools, for building a unified, prevention-oriented paradigm. A real-world case study on bisphenol A (BPA) substitution illustrates the benefits in practice. Finally, policy and research implications are discussed. Overall, the aim is to show how combining life-course exposure insights with a life-cycle SSbD approach can better protect people and the environment from avoidable chemical harms.

## Conceptual underpinnings of SSbD

SSbD builds on green chemistry principles to minimise hazardous substances, improve energy efficiency, prioritise renewable feedstocks and favour products that degrade benignly [8]. These principles were subsequently expanded through life-cycle thinking and a more explicit precautionary framing [4, 9]. SSbD incorporates this foundation but goes further by integrating hazard and exposure considerations into every stage of innovation, from early design choices through production and market deployment [2, 10]. It shifts the focus from optimising individual industrial processes to examining the full chain from raw material extraction and manufacturing to use, recycling and disposal [11], while also acknowledging social dimensions such as fair labour, reduced environmental injustice around production sites and transparency along supply chains [3].

SSbD aims to tackle risks at the source. Chemists, chemical engineers and product developers are encouraged to identify and minimise, or ideally eliminate, intrinsic hazards before products reach the market, rather than relying on downstream controls [4]. Safety is therefore embedded at the molecular, process and product levels; for example, selecting a polymerisable monomer that is low-toxicity, non-bioaccumulative and able to degrade safely can substantially reduce the need for protective measures later in the life cycle [12]. A second core tenet is rigorous life-cycle assessment, framed as cradle-to-grave or cradle-to-cradle analysis [13]. Each stage is evaluated in terms of resource use, waste generation and potential exposure pathways to avoid burden shifting, such as reducing toxic releases during manufacturing while generating persistent hazardous wastes at end-of-life [5].

SSbD is closely aligned with circular economy concepts, favouring designs that enable reuse, recycling or safe biodegradation [2, 14]. Engineering materials to be disassembled or broken down via benign pathways reduce environmental impacts and associated risks. Implementation faces barriers: data gaps on safer alternatives [15], rapid innovation in areas such as nanotechnology relative to hazard assessment [16] and economic incentives that privilege legacy chemicals, often requiring policy measures including the European Commission’s Chemicals Strategy for Sustainability [2]. Seen through a life-course exposome lens (Wright and Hanson 2022), SSbD offers a proactive framework for reducing exposures across the chemical life cycle, while underscoring the need for robust epidemiological and exposomic evidence to guide design decisions.

## The life-course exposome approach

The exposome, first articulated by Wild, expands disease causation beyond the genome to environmental exposures across the life-course [1]. Multiple external stressors interact with genetic susceptibility to shape risk [17, 18], yet this complexity is largely missed by conventional risk assessment, which typically evaluates only a few chemicals at a time [19]. A life-course view also recognises that timing is critical: exposures during fetal development, childhood or adolescence can induce long-lasting effects that manifest decades later [6, 20]. Social and structural determinants such as deprived neighbourhoods, discrimination and precarious employment further modulate exposure profiles and vulnerability [21, 22].

**Fig. 1:**
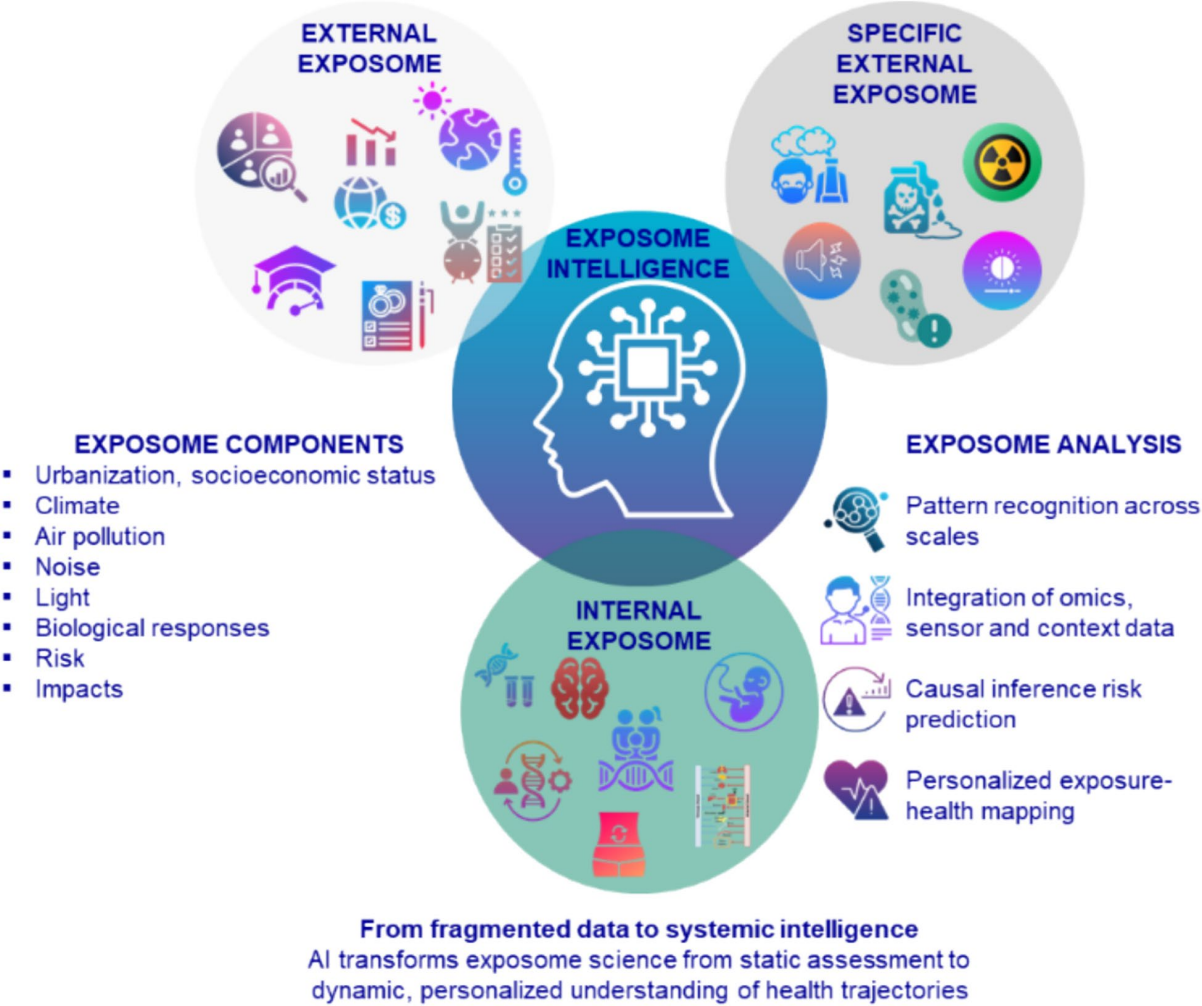
Key exposome components

Externally, the exposome spans environmental and occupational pollution, consumer and household products, industrial emissions and diet [22]. Internally, it appears as physiological, molecular and biochemical responses to these stressors, captured through biomarkers in biological samples that quantify body burdens and pathway perturbations [23, 24]. Advances in omics technologies allow systematic characterisation of how complex exposure profiles reconfigure molecular networks over time [18, 25]. Mapping exposure trajectories and molecular signatures across critical windows helps identify mixtures that contribute most to lifelong risk of outcomes such as cardiovascular disease and cancer [26, 27] (Fig. 1).

Recent exposome research has converged on an integrative toolbox linking what people encounter to what their biology experiences [28]. High-resolution mass spectrometry expands the detectable chemical space [29], while exposome-wide association studies scan exposure–phenotype relationships at scale [30–32]. Wearable and personal sensors characterise micro-environments using geo-located, real-time environmental and physiological data [21, 33]. Coupling these external measurements with physiologically based biokinetic (PBK) models and multi-omics readouts enables tracing how mixtures penetrate the body, perturb pathways and create windows of susceptibility along the life-course [18, 23, 34]. This integration bridges “what is out there” with “what gets inside and matters” and provides a mechanistic basis on which SSbD can anticipate risk rather than react to it [35].

**Fig. 2:**
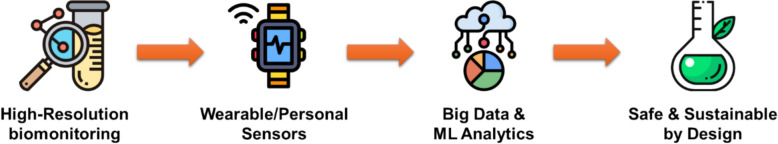
Methodological advances in exposome research

The resulting data-rich landscape demands advanced analytics. Machine-learning and related approaches detect non-linear patterns, exposure clusters and gene–environment interactions [30, 36]. By highlighting substances and mixtures that disproportionately affect vulnerable populations and developmental windows, exposomic evidence guides SSbD towards redesigning molecules, production processes and product formulations, helping to avoid regrettable substitutions [37]. More broadly, the convergence of wearable technologies, biomonitoring and integrative data science underscores the value of the exposome paradigm for understanding environmentally driven biochemical perturbations [38] and, when aligned with design-for-prevention strategies, for supporting healthier, more sustainable environments [39] (Fig. 2).

## Points of convergence between SSbD and the life-course exposome approach

**Fig. 3:**
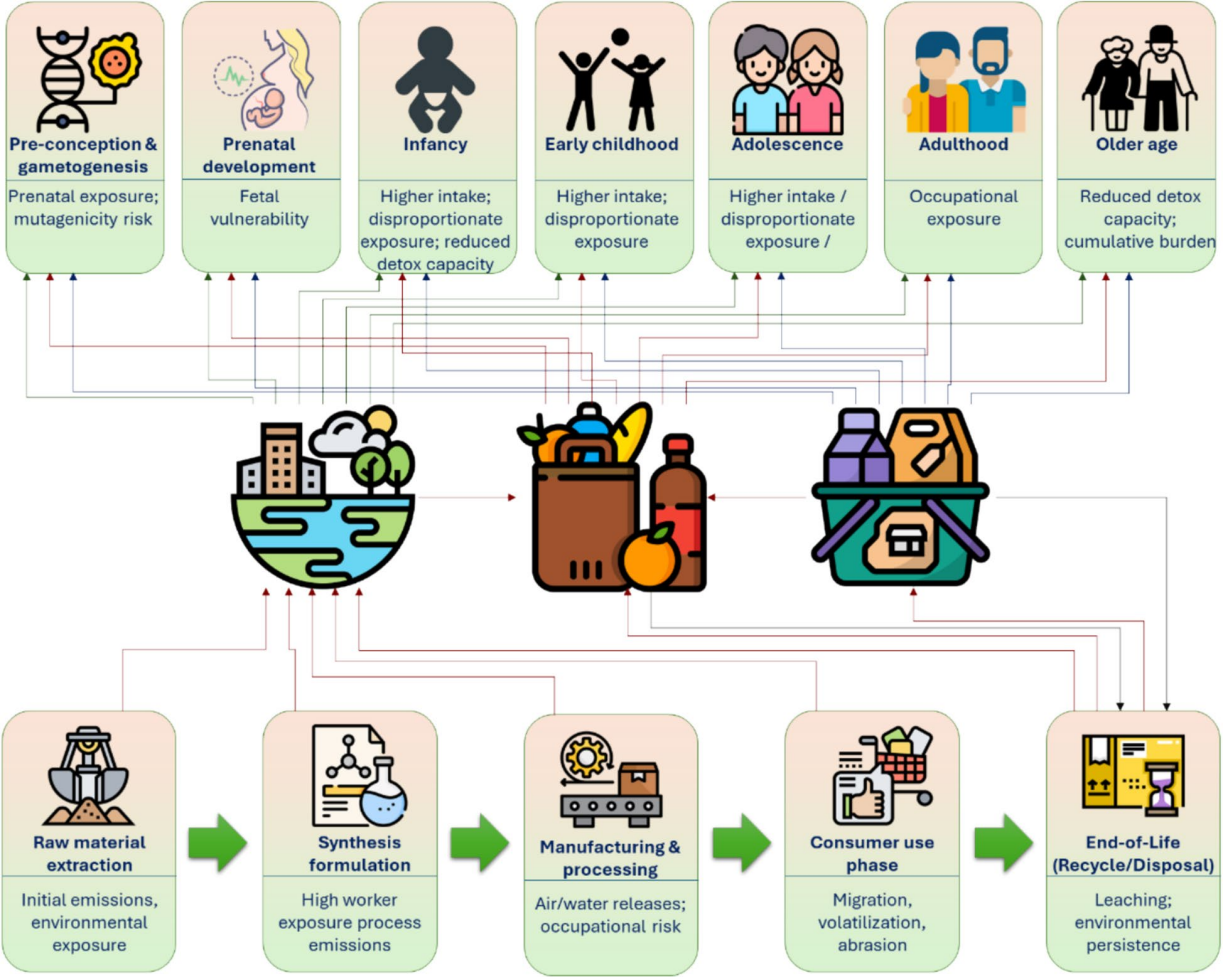
Linking product life cycle and human life course through exposure pathways

**Fig. 4:**
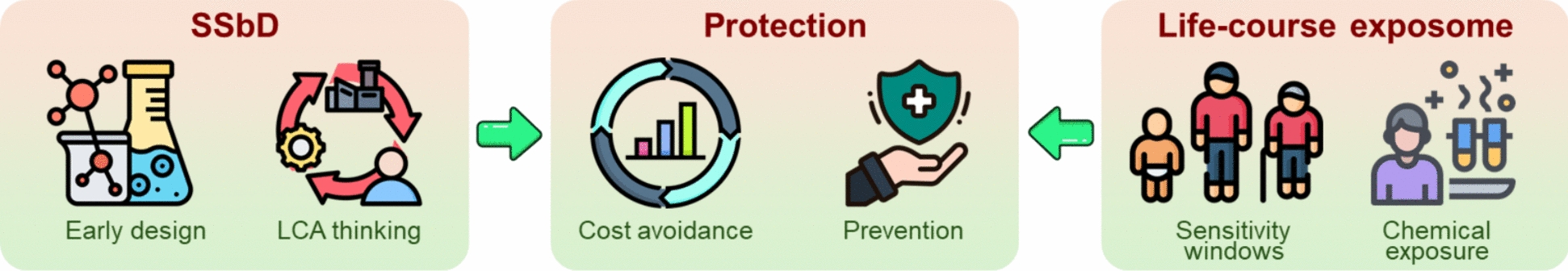
Key points of convergence between SSbD and the life-course exposome approach

Both SSbD and the life-course exposome perspective emphasise acting early and at the right points in time to prevent harm. SSbD maps a product’s life cycle (Fig. 3), from raw material extraction through synthesis, use and end-of-life, to identify where hazardous substances may escape to the environment or reach people, and seeks to remove or substitute those compounds before they are widely used [17, 40, 41]. Chemicals that interfere with neurodevelopment, immune function or reproduction are therefore priority targets for elimination or strict control during design, aligning SSbD choices with preventive health goals [6]. These shared temporal perspectives are summarised in Fig. 4, which links product life-cycle stages to human life-course vulnerabilities.

A second major point of convergence is the treatment of chemical mixtures. Real-world exposures rarely involve single substances; people experience complex cocktails via air, water, diet and consumer products, with cumulative and sometimes synergistic toxicity [19, 40]. Conventional risk assessment often considers compounds in isolation, overlooking mixture effects [42]. SSbD responds by accounting for co-formulants, by-products and contaminants across the life cycle, aiming to reduce both single-substance hazards and the overall burden from combined exposures [12]. The life-course exposome approach complements this by characterising the full spectrum of exposures in everyday settings [22] and examining how multiple agents interact [43]. Biomonitoring studies routinely detect multiple chemicals in human tissues [44], sometimes at levels or in combinations that raise concern, especially in sensitive subgroups [7]. Using these data to inform SSbD enables early identification of problematic mixtures and supports more holistic regulation aimed at lowering cumulative toxicity [45].

Equity and environmental justice represent a further area of alignment. Populations in lower socioeconomic status areas, often located near industrial facilities or dense traffic, experience disproportionate exposure to air pollution, hazardous waste and chemical releases [46]. The life-course exposome clarifies how these unequal burdens accumulate and are amplified during critical periods such as pregnancy, infancy and adolescence, contributing to health disparities [47]. SSbD offers a forward-looking response by shifting responsibility upstream to manufacturers and policymakers, who are urged to redesign products and processes to reduce toxicity and resource use, thereby preventing harms before they reach communities [5]. Exposomic evidence helps identify chemicals and use patterns that disproportionately affect disadvantaged groups and prioritise them for substitution or phase-out within SSbD strategies. In combination, these approaches aim not only to reduce overall exposure but also to narrow chemical-related health inequalities and support sustainable environments and development through innovation.

Finally, an emerging convergence lies in data integration and cross-disciplinary evidence. Both SSbD and exposomics generate diverse datasets, from chemical properties, production volumes and life-cycle emissions to biomonitoring results, omics signatures and sensor-based micro-environment measurements. Establishing interoperable data structures and common metrics allows life-cycle information to feed exposure and risk models, while exposomic findings feed back into design and substitution decisions [38]. This cross-cutting convergence requires sustained collaboration among chemists, engineers, toxicologists, exposure scientists and public health researchers, and accelerates the translation of exposomic insights into safer product design, ultimately advancing the shared preventive goals of both frameworks.

## Methodological integration toward a unified framework

The practical link between life-cycle assessment and the life-course exposome is a data translation challenge: moving from inventory flows (emissions, uses, scenarios) to intake, internal dose and pathway perturbation. A tractable strategy is a two-step mapping. First, life-cycle emissions and product-use scenarios are converted into population and micro-environmental exposures using fate and exposure models. Second, quantitative in vitro–to–in vivo extrapolation (qIVIVE) and PBK modelling infer tissue doses that can be directly compared with New Approach Methodology (NAM)-derived bioactivity points of departure on relevant Adverse Outcome Pathways (AOPs) [23]. This harmonization allows SSbD to test whether internal dose distributions under realistic conditions overlap with biologically relevant effect levels derived from NAM- and AOP-anchored endpoints and, where they do, to trigger redesign or restriction.

Next-Generation Risk Assessment (NGRA) for SSbD is explicitly human-relevant and exposure-led: it starts from realistic use scenarios, mechanistic hazard characterization and explicit treatment of variability and mixtures [37, 48]. Rather than assessing one chemical at a time, NGRA couples population exposure distributions to AOP-anchored potency metrics from NAMs and examines sensitive subgroups and life-stage windows. If predicted internal doses for, e.g., infants, pregnant workers or the elderly approach effect levels on critical AOPs, structure or use conditions must change before innovation proceeds [49]. Vulnerability reflects intrinsic factors (genetics, age, pre-existing disease) and extrinsic stressors (co-exposures, social determinants) that shift dose–response [50]. For SSbD, these are design constraints: structures and uses should be chosen so that even upper-percentile doses in vulnerable windows remain below AOP-linked thresholds, penalising features that drive persistence or bioaccumulation, limiting volatility that increases inhalation, and avoiding moieties tied to endocrine or neurodevelopmental pathways [51].

Toxicodynamics connects molecular features to cascades from target engagement to adverse outcomes. AOPs formalise these cascades, linking molecular initiating events to key events and outcomes, increasingly in quantitative form [52]. For SSbD, AOP networks and systems-toxicology models become design maps: substructures that trigger well-mapped AOPs (e.g. electrophiles driving oxidative stress or fibrotic pathways) are avoided, while scaffolds not engaging vulnerable pathways are favoured. NAM readouts can be projected onto AOP networks to flag mechanism-related risk early, and structure alerts can be refined as AOP knowledge grows [53].

**Fig. 5:**
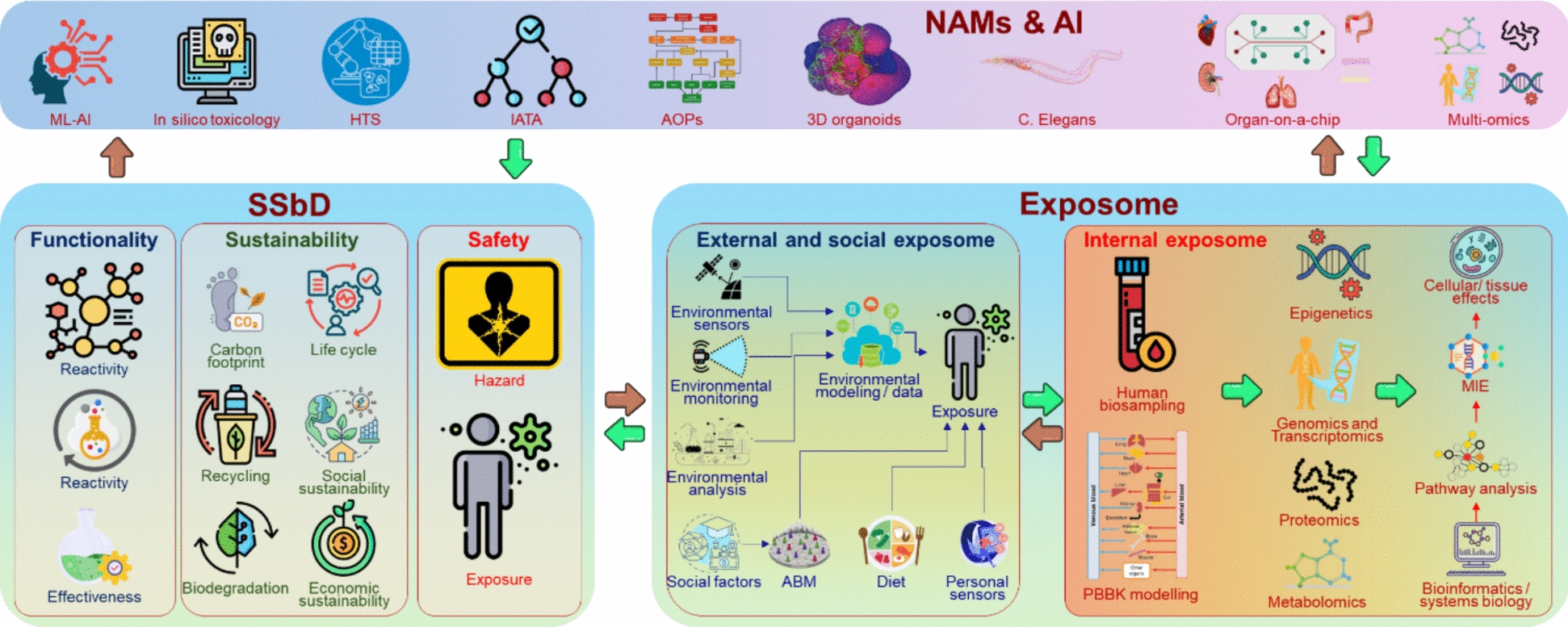
Common feedback loops between exposomics, SSbD and next-generation risk assessment (NGRA)

Multi-omics platforms provide dense, pathway-level fingerprints that anticipate adversity well before apical effects emerge. High-throughput transcriptomics and high-content cell-morphology profiling identify which stress-response circuits and developmental programmes are perturbed [54], while metabolomics captures shifts in energy, lipid and signalling pathways central to development and cardiometabolic disease [55]. When these omics responses are benchmark-dosed and anchored to AOP key events, they become quantitative design constraints: candidates that activate endocrine, neurodevelopmental or fibrotic signatures at low internal concentrations are deprioritized; those with largely quiescent omics profiles at relevant doses move forward, subject to exposure and degradability checks (Fig. 5).

NAMs weave together in silico predictions, high-throughput and high-content bioassays, targeted in vitro kinetics and microphysiological systems into coherent, human-relevant evidence streams [49]. Their value for SSbD is two-fold: they enable “fail-fast” screening before synthesis or scale-up, and they mechanistically anchor safety, allowing qIVIVE and PBK models to link external use conditions to internal effect levels. Confidence is being strengthened through formal validation and Good In Vitro Method Practices, with regulatory acceptance expanding where methods are fit-for-purpose [56]. Exposome data then close the loop between prediction and reality: human biomonitoring and environmental surveillance test whether “safe-by-design” chemicals remain below internal levels of concern, while non-target screening detects unexpected metabolites or co-occurring mixtures [57]. When exposure drifts upward or omics biomarkers indicate pathway engagement in sentinel groups, PBK [23] and AOP context [58] explain why, and design or use conditions are iteratively adjusted [48]. Mechanistic NAMs, multi-omics and life-course exposomics thus form a systems-biology feedback loop that keeps SSbD protective across the life-course, not just on paper.

Achieving this integration requires regulatory frameworks that encourage or require pre-market omics and NAM testing. Although many reviews still focus on single substances, agencies such as the US EPA and ECHA are beginning to integrate high-throughput in vitro data and mechanistic evidence into decision-making [59, 60]. Programs like Tox21 are further refining high-throughput screening and organ-on-chip methods to capture early, life-stage-specific toxicity markers. As these technologies mature, SSbD practitioners can rely on them to design hazards out of products and processes before risks become widespread, aligning chemical innovation with primary prevention.

Within this framework, decision-support tools such as multi-criteria decision analysis (MCDA) help navigate trade-offs among function, safety and sustainability [15, 61]. MCDA combines hazard metrics, life-stage-specific risks, life-cycle indicators (resource use, carbon, water) and exposomic relevance, including whether substances accumulate in humans or vulnerable groups [30, 62, 63]. Exposome-informed MCDA thus guides SSbD choices toward options that minimize real-world exposure while meeting performance and societal needs, operationalizing systems-level evidence into concrete design decisions.

## Real-world illustrations and case study

### Introduction

The phase-out of bisphenol A (BPA) in polycarbonate (PC) plastics provides a clear example of how SSbD converges with exposome-based thinking [64, 65]. For decades, BPA was a key monomer because it combined durability, clarity and heat resistance in products ranging from food containers to medical devices [66]. Subsequent evidence showed that BPA can mimic estrogen and act as an endocrine disruptor [67], and it has been increasingly detected in the environment, raising concerns about long-term human and ecological effects [68, 69]. Biomonitoring indicates that exposure begins in utero and continues through childhood, with infants and children especially vulnerable [70].

### Methods

We conducted a comparative SSbD case study of BPA, BPAP, and isosorbide in reusable PC bottles, following the SSbD framework assessment workflow (hazard, exposure, environmental sustainability) with a life-cycle perspective. Hazard evaluation combined literature data and in silico predictions (e.g. QSARs) to screen toxicity endpoints. Exposure assessment used existing **exposome data** and modeling: for BPA we drew on human biomonitoring and typical use scenarios (2 L/day water intake from a PC bottle), while for BPAP and isosorbide we estimated upper-bound migration into water and used Monte Carlo exposure models (e.g. INTEGRA by Sarigiannis, Karakitsios [71]) during production and use. Environmental sustainability was assessed via cradle-to-grave LCA: we compared environmental footprints (e.g. global warming potential, resource use) of producing and using PC bottles made from each monomer. Within the operationalization context of SSbD, the European Partnership for the assessment of risks from chemicals (PARC; Marx-Stoelting, Rivière [72]) is developing the PARC SSbD toolbox [73]. This toolbox is designed to be an integrated platform of tools, including methods, models, algorithms and data sources, for the practical implementation of the SSbD framework. In the BPA case, where possible, we referenced findings from the PARC SSbD toolbox testing (case study data) and published studies [74].

### Results

#### Hazard profiles

BPA exhibits well-documented endocrine activity [75, 76] and multiple toxic effects. It binds estrogen receptors, is linked to reproductive and metabolic disorders, and is classified with hazards for developmental toxicity. BPAP, due to its bisphenol structure, is predicted to be similarly endocrine-active. In fact, expert assessments conclude that BPAP (like BPS) is so chemically similar to BPA that “substitution would probably not lead to significant health benefits”. By contrast, isosorbide differs fundamentally: it is a sugar-based bicyclic diol without BPA’s phenolic rings. Published reports [77, 78] emphasize that isosorbide-based PC is “*well-known for its … nontoxic properties*”. No endocrine or chronic toxicity has been reported for isosorbide itself, and in silico screening yields no structural alerts for hormone disruption. Thus, from a hazard standpoint, the ranking is BPA ≈ BPAP >  >  > isosorbide.

#### Exposure assessment

Exposure assessment for BPA, BPAP, and isosorbide in the polycarbonate reusable bottle application was conducted under early innovation conditions following the SSbD framework. Predicted environmental concentrations (PECs) and consumer exposure estimates were derived using INTEGRA [71], complemented by migration modelling with Vermeer FCM for the use phase. Conservative assumptions and QSAR-derived physicochemical and fate parameters were applied to reflect limited data availability at this stage. During manufacturing and processing, BPA and BPAP exhibited relatively high predicted regional PECs, particularly in freshwater and marine water compartments, with concentrations on the order of 10^2^ μg/L. These PECs resulted in screening-level environmental risk characterization ratios (RCRs) exceeding unity, indicating potential environmental concern under worst-case assumptions. Soil and sediment RCRs were substantially lower but still non-negligible for bisphenols. In contrast, isosorbide showed PECs several orders of magnitude lower across all environmental compartments, leading to negligible RCRs. This difference reflects both lower environmental persistence and substantially higher predicted no-effect concentrations for isosorbide, even when conservative QSAR-based values are applied. During use phase (consumer exposure), exposure via migration from polycarbonate bottles was predicted to be very low for all substances. Resulting consumer RCRs were well below unity for BPA and BPAP under early innovation assumptions, largely driven by low migration fractions and physicochemical constraints. However, these outcomes remain sensitive to the toxicological thresholds applied, particularly for BPA. Isosorbide again displayed the lowest exposure profile, with predicted consumer exposure several orders of magnitude lower than for bisphenols and negligible RCRs. Overall, the exposure assessment highlights substantially higher environmental exposure potential for BPA and BPAP during production, while isosorbide consistently demonstrates minimal environmental and consumer exposure in the polycarbonate application (Table 1).

**Table 1:** PEC and intake levels for the three alternatives, under the polycarbonate exposure scenario

Chemical	SSBD STEP	Exposure domain	Pathway/medium	Metric	Value	Units
BPA	Manufacturing and processing	Environment (regional)	Freshwater	PEC	1.36∙10^2^	µg/L
			Marine water		4.33∙10^1^	µg/L
			Freshwater sediment		2.07	µg/kg dw
			Soil		5.31∙10^1^	µg/kg dw
	Step 3	Consumer use	Water/beverages (ingestion)	Daily mean intake	6.0∙10⁻^3^	µg/kg_bw_/day
BPAP	Manufacturing and processing	Environment (regional)	Freshwater	PEC	1.54∙10^2^	µg/L
			Marine water		5.41∙10^1^	µg/L
			Freshwater sediment		2.32	µg/kg dw
			Soil		5.07∙10^1^	µg/kg dw
	Use phase	Consumer use	Water/beverages (ingestion)	Daily mean intake	4.0∙10⁻^3^	µg/kg_bw_/day
ISOSORBIDE	Manufacturing and processing	Environment (regional)	Freshwater	PEC	4.24∙10⁻^11^	µg/L
			Marine water		1.91∙10⁻⁹	µg/L
			Freshwater sediment		3.61∙10⁻^21^	µg/kg dw
			Soil		5.15∙10⁻^14^	µg/kg dw
	Use phase	Consumer use	Water/beverages (ingestion)	Daily mean intake	9.0∙10⁻^3^	µg/kg_bw_/day

#### Life-cycle sustainability

Life cycle analysis (LCA) contrasting BPA- vs isosorbide-based PC bottles reveals clear sustainability differences. Isosorbide is bio-derived (from starch or cellulose) and its PC resins have been designed for recyclability, whereas BPA-PC is a petrochemical product and falls into mixed “other” plastics recycling codes. LCA results showed that an isosorbide-PC bottle generally **lowers global warming potential** relative to BPA-PC. For example, because isosorbide-PC can match the thermal performance of PET (allowing PET-recycling infrastructure), the net carbon footprint per bottle is reduced. Some impact categories, however, increase due to the biobased route: isosorbide production demands more water and can raise acidification/water use impact on that stage. Overall, though, the **integrated environmental footprint (EF)** was lower for isosorbide-PC (driven largely by a smaller production footprint in fossil terms). By contrast, the life cycle impact of BPA-PC was dominated by its fossil fuel-based production. BPAP would track with BPA on the use of fossil resources (it is synthetically made and not renewable). Thus, environmental sustainability metrics combining eco-toxicity, carbon emissions, and resource use of fossils over the chemical life cycle favor isosorbide over BPA or BPAP.

#### Integrating life-course–relevant bioactivity evidence into early-stage safe and sustainable by design (SSbD) decision-making

To demonstrate the feasibility of integrating life-course–relevant bioactivity evidence into early-stage Safe and Sustainable by Design (SSbD) decision-making, the three candidate chemicals, were evaluated using an identical, pathway-anchored high-throughput screening (HTS) and physiologically based pharmacokinetic (PBPK) framework. For all three substances, ToxCast/Tox21 assay-level data were retrieved from the US EPA CompTox Dashboard as a starting point and curated using the same pre-specified selection rules (pathway-anchored filtering), to ensure full methodological symmetry and enabled direct comparison of bioactivity-derived benchmarks at early stages of innovation, as described below:

- Rule 1 (target relevance): retain assays annotated to the intended molecular targets (primary: ERα/ERβ transactivation/response element assays; secondary: TSHR assays in parallel).
- Rule 2 (signal quality): retain only assays with interpretable concentration–response behaviour (exclude endpoints flagged as non-informative or dominated by technical artefacts; document curve quality/flags where available).
- Rule 3 (cytotoxicity confounding): deprioritise or exclude endpoints consistent with non-specific cytotoxicity (e.g., “cytotoxicity burst” patterns) unless used explicitly as a context flag.
- Rule 4 (bioactivity anchor definition): define the ER anchor as the most sensitive (lowest AC50) retained ER assay with adequate signal quality; treat TSHR activity as a supportive parallel line of evidence rather than the anchor for reverse dosimetry.
- Rule 5 (transparency): report retained assays, AC50 values, maximum tested concentrations, and inclusion/exclusion reasons in Table/Supplement.

BPA exhibited robust ER-mediated bioactivity across multiple mechanistically interpretable assays, including ERα transactivation and estrogen response element reporter endpoints. After exclusion of background measurements and non-specific cytotoxicity-associated responses, a consistent ER signal remained, allowing conservative identification of an ER anchor concentration (AC50*) within the tested concentration range. TSHR-related activity was also observed for BPA, but only at substantially higher concentrations, confirming that estrogenic signaling represents the dominant endocrine perturbation pathway. The retained ER AC50* was subsequently propagated through PBPK-based reverse dosimetry to derive bioactivity-informed external dose benchmarks suitable for comparison with predicted internal exposure distributions.

BPAP, evaluated using the same HTS evidence stream and filtering logic, showed a qualitatively similar but quantitatively distinct profile. As with BPA, ER-mediated bioactivity was retained following pathway-anchored curation, with the most sensitive ERα transactivation endpoint providing a conservative AC50* for reverse dosimetry. Parallel evaluation of TSHR assays indicated thyroid-related activity only at much higher concentrations, reinforcing ER signaling as the primary driver of potential endocrine concern. Importantly, the ER anchor for BPAP was of comparable magnitude to that of BPA, supporting concerns regarding potential regrettable substitution when replacing BPA with structurally similar bisphenols based solely on hazard class substitution or life-cycle considerations.

In contrast, isosorbide displayed a fundamentally different HTS profile. Application of the same ER and TSHR selection rules resulted in no retained endocrine effect endpoints after exclusion of background, channel-only, and cytotoxicity-associated responses. ERα, ERβ, and TSHR assays were inactive up to their respective maximum tested concentrations. Rather than interpreting this absence of activity as evidence of safety, a conservative bound-based approach was adopted: the highest tested concentration with no observed effect was treated as a lower bound on the internal concentration required to elicit endocrine pathway perturbation. Propagation of this bound through PBPK reverse dosimetry yielded bioactivity benchmarks that substantially exceeded predicted internal exposures under realistic use scenarios.

In exposome research, internal dose metrics such as peak (C_max_) and steady-state (C_ss_) plasma concentrations are essential for comparing biologically relevant exposures across chemicals. Bisphenol A (BPA) is rapidly absorbed following oral intake but undergoes extensive first-pass metabolism in humans, predominantly via glucuronidation [79, 80]. As a result, the systemic bioavailability of parent BPA is very low [65]: more than 90% of an ingested dose is converted to BPA-glucuronide and excreted in urine within 24 h [79]. The terminal elimination half-life of BPA is approximately 6 h, and unconjugated BPA typically represents less than 1% of total circulating BPA [80]. Consequently, BPA exposure is characterized by a transient nanomolar-range C_max_ of the free compound and negligible accumulation under once-daily dosing, resulting in an effectively negligible C_ss_ [81]. Bisphenol AP (BPAP), a structural analogue of BPA, is expected to exhibit even lower systemic exposure at equivalent oral doses. This is primarily attributable to its poor oral absorption, related to low aqueous solubility (~ 1 mg/L) and higher lipophilicity (log K_ow_ ≈ 4.9) [82]. The absorbed fraction of BPAP is anticipated to undergo rapid phase II metabolism similar to BPA, mainly glucuronidation [83]. Although its higher lipophilicity may increase tissue distribution and marginally prolong elimination, BPAP is still expected to reach a lower plasma C_max_ than BPA and not to achieve substantial steady-state concentrations. Direct human pharmacokinetic data for BPAP remain limited, and these conclusions rely on structural analogy and available in vivo metabolite evidence [84]. In contrast, isosorbide, a sugar-derived BPA alternative, displays a markedly different pharmacokinetic profile. It is highly water-soluble, well absorbed orally, and shows minimal first-pass metabolism [85]. Isosorbide distributes primarily within total body water (volume of distribution ~ 0.6 L/kg), does not bioaccumulate in adipose tissue, and is rapidly eliminated via renal excretion, largely as conjugated metabolites, with a plasma half-life of only a few hours [86]. Consequently, for an equivalent mg/kg/day oral dose, isosorbide may exhibit a higher initial C_max_ of the parent compound but maintains a low C_ss_ due to efficient clearance. Overall, these differences illustrate how modest structural variations translate into pronounced differences in ADME behavior and internal exposure, underscoring the importance of internal dose metrics in exposome-based assessments [23].

**Fig. 6:**
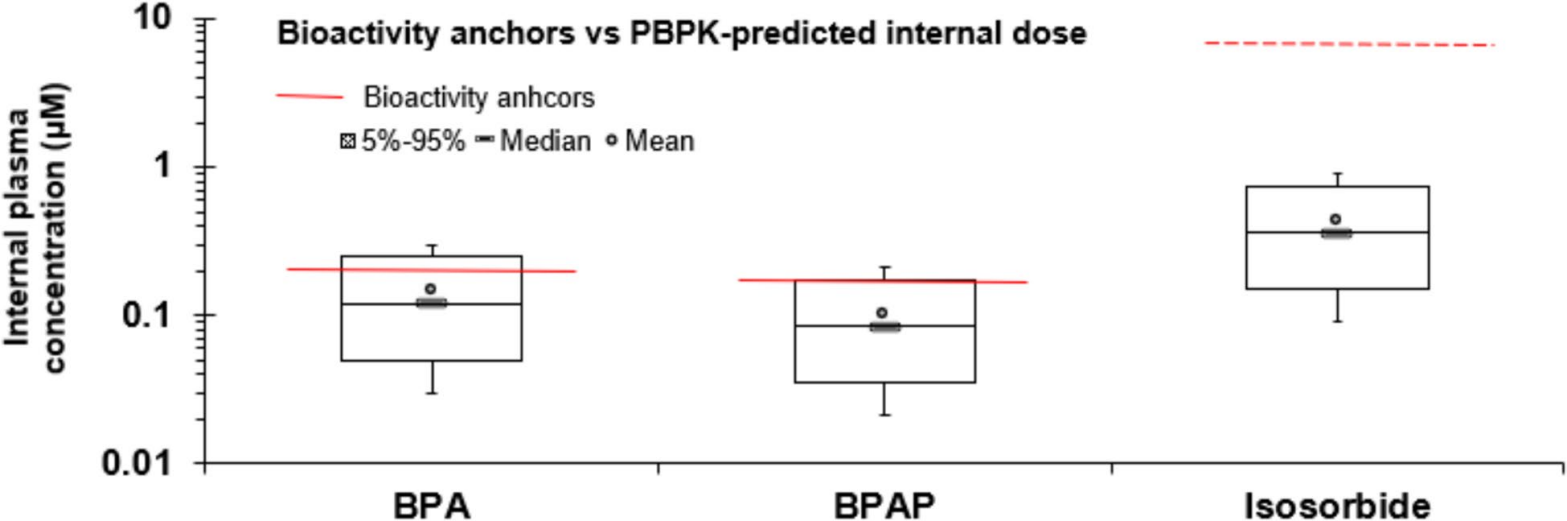
Integration of ToxCast ER bioactivity anchors with PBPK-predicted internal exposure for SSbD stage-gate decision-making: Estrogen receptor (ER) bioactivity anchors (horizontal lines) derived from pathway-anchored ToxCast assay selection (AC50*) are shown in relation to PBPK-predicted internal plasma concentration ranges (vertical whisker plots) under realistic use-phase exposure scenarios for BPA, BPAP, and isosorbide. For BPA and BPAP, predicted internal exposures overlap ER bioactivity anchors, indicating insufficient safety margin and failure of the early SSbD safety stage-gate. In contrast, isosorbide shows no retained ER activity up to the maximum tested concentrations (dashed bound), with PBPK-predicted internal exposures remaining well below this bound, indicating passage of the early SSbD safety gate. This one-panel integration operationalises PARC SSbD Toolbox logic by linking Tier-1 hazard screening with Tier-2 exposure modelling at early innovation stages

The overall safety assessment, integrating real-life exposure regimes—PBPK predicted internal dose and bioactivity anchors over the life course is presented in Fig. 6. The figure compares PBPK-predicted internal plasma concentration distributions with ER bioactivity anchors derived from ToxCast assays, providing a direct SSbD stage-gate perspective. For BPA and BPAP, the median and upper-percentile internal concentrations overlap or closely approach the ER bioactivity anchors, indicating limited or absent safety margins under realistic use-phase exposure scenarios. This convergence suggests that both substances remain capable of eliciting estrogen receptor–mediated effects at internally relevant doses, supporting their classification as failing the early SSbD safety gate and highlighting the risk of regrettable substitution in the case of BPAP. In contrast, isosorbide displays internal concentration distributions several orders of magnitude below its conservative bioactivity bound, indicating a robust margin of safety and supporting progression beyond the early SSbD stage-gate.

The ER bioactivity anchors used in this analysis correspond to a well-established molecular initiating event (MIE) within estrogen receptor–mediated adverse outcome pathways (AOPs), linking receptor activation to downstream key events such as altered gene expression, endocrine disruption, and reproductive and developmental effects. By combining PBPK-predicted internal concentrations with ER-specific bioactivity thresholds, the results operationalise an AOP-informed decision logic: overlap between internal dose distributions and the ER MIE activation threshold indicates a non-negligible probability of triggering downstream key events along the AOP. Accordingly, BPA and BPAP show insufficient margins at the MIE level, implying plausible propagation toward adverse outcomes. In contrast, isosorbide remains far below the ER MIE activation bound, suggesting a low likelihood of initiating the corresponding AOP cascade under realistic exposure scenarios.

### Discussion

**Fig. 7:**
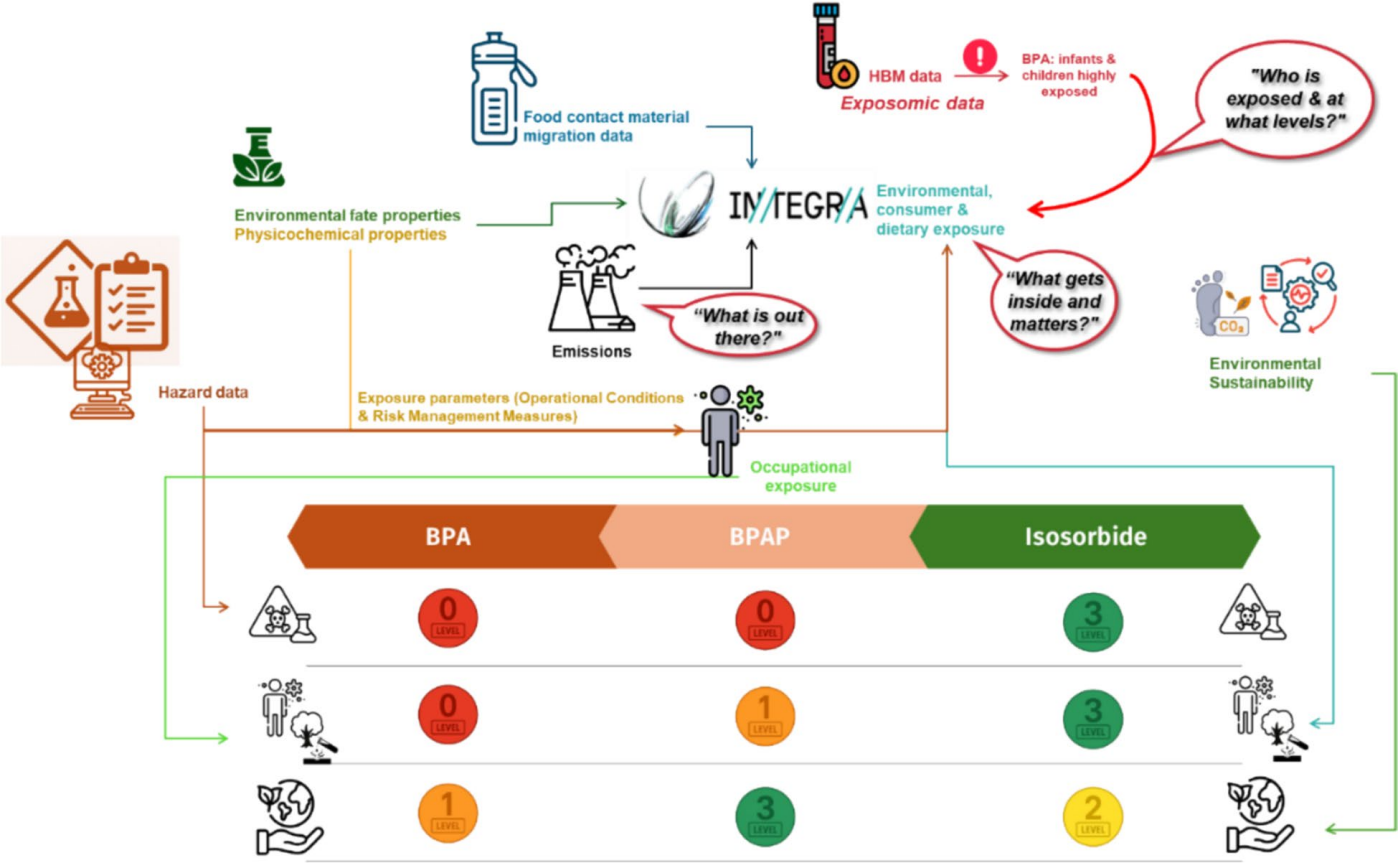
Methodological workflow and results of the BPA case, including the SSbD profile of each chemical for the dimensions of hazard, risk of exposure and environmental sustainability. An SSbD level of 0 reflects red flags with a poor SSbD profile, while levels gradually increase (from level 0 to levels 1 and 2) up to level 3, which indicates a beneficial SSbD profile

This case study demonstrates that, when substitution of bisphenol A (BPA) in reusable polycarbonate (PC) bottles is evaluated through an integrated life-course exposome–SSbD lens, isosorbide clearly emerges as the preferred alternative, whereas bisphenol AP (BPAP) exemplifies a likely regrettable substitution. Across hazard, exposure, and environmental sustainability dimensions, isosorbide consistently outperforms both BPA and BPAP within the PARC SSbD toolbox, achieving higher SSbD maturity levels and passing early safety stage-gates that the bisphenols fail. As synthesised in Fig. 7, isosorbide attains “3–3–2” SSbD levels for hazard, processing, and use in the late-innovation stage following the framework of Caldeira et al. (2023), while BPA and BPAP remain constrained at level 0 or 1 on at least one axis. These differences reflect fundamental structural distinctions between aromatic bisphenols and aliphatic, sugar-derived monomers, with direct implications for endocrine bioactivity, exposure potential, and life-cycle impacts.

As shown in Table 2 and Fig. 6, ER-mediated bioactivity translated via PBPK provides a clear early SSbD stage-gate signal: BPA and BPAP fail due to overlap between internal exposure and MIE thresholds, whereas isosorbide shows a robust margin of safety. ER transactivation/response-element assays provide evidence for estrogen receptor activation, which can be treated as a molecular initiating event or early key event within ER-mediated AOPs. For example, breast cancer AOP development work explicitly frames ERα activation and downstream mechanistic changes in AOP terms and maps these to non-animal methods [87]. PMC In addition, endocrine-disruption AOP work on uterine adenocarcinoma highlights ER activation in uterine tissue as a critical node within a putative AOP network, supporting the use of ER-anchored NAM evidence for early screening decisions when life-course windows are central.

**Table 2:** Key ToxCast endocrine assays retained after pathway-anchored filtering and corresponding AC50 values: Assays retained following exclusion of background/channel endpoints and nonspecific cytotoxicity-associated responses

Chemical	Assay name	Biological target/pathway	Endpoint type	AC50 (µM)	Max tested conc. (µM)	Role in SSbD decision
***BPA***	ATG_ERa_TRANS	ERα signaling	Transactivation	sub-µM–low µM	within tested range	**Primary ER anchor (fail safety gate)**
*BPA*	ATG_ERE_CIS	ERα signaling	Reporter gene (ERE)	low µM	within tested range	Supporting ER evidence
*BPA*	TOX21_TSHR_HTRF_Agonist_ratio	Thyroid signaling (TSHR)	Agonist ratio	tens of µM	within tested range	Parallel endocrine line (secondary)
***BPAP***	**ATG_ERa_TRANS**	**ERα signaling**	**Transactivation**	**0.145**	**0.823**	**Primary ER anchor (fail safety gate)**
*BPAP*	ATG_ERE_CIS	ERα signaling	Reporter gene (ERE)	0.18	7.41	Supporting ER evidence
*BPAP*	TOX21_ERb_BLA_Antagonist_ratio	ERβ signaling	Antagonist ratio	~ 0.30*	0.0026	Supportive only (AC50 > max tested)
*BPAP*	TOX21_TSHR_HTRF_Agonist_ratio	Thyroid signaling (TSHR)	Agonist ratio	59.1	92.2	Parallel endocrine line (secondary)
***Isosorbide***	ERα/ERβ assays (multiple)	ER signaling	Various	** > max tested**	assay-specific	**No retained ER activity (pass safety gate)**
*Isosorbide*	TSHR assays (multiple)	Thyroid signaling	Various	** > max tested**	assay-specific	No retained thyroid activity

Estrogen receptor (ER) signaling treated as primary SSbD hazard hypothesis; thyroid stimulating hormone receptor (TSHR) evaluated in parallel

The life-course exposome perspective provides the “who–what–when” context that gives these mechanistic findings practical relevance. Biomonitoring data indicate that BPA exposure is widespread and persistent, with infants and children using PC drinkware representing a highly exposed and vulnerable subgroup [65]. Anchoring SSbD decisions to internal dose distributions over the life course, rather than intrinsic hazard alone, sharpens the design question from *“Is this molecule less hazardous in principle?”* to *“Does this redesign meaningfully reduce internal exposures during critical windows of susceptibility?”* For BPA and BPAP, the answer remains negative; for isosorbide, the answer is provisionally affirmative.

Figure 7 situates these findings within the broader SSbD workflow, linking PBPK–HTS–AOP evidence with life-cycle inventory data, exposure scenarios, and sustainability metrics. Importantly, the interaction between exposome science and SSbD is bidirectional. While exposomic evidence prioritises substances and uses for redesign, the SSbD framework, implemented through the PARC toolbox, provides a structured, stage-gated decision architecture that translates complex exposure and bioactivity data into actionable design constraints. Explicit definition of functional units, use descriptors (ERCs, PROCs), production volumes, and life-cycle boundaries disciplines exposome modelling and ensures that exposure estimates remain anchored to realistic innovation contexts.

**Fig. 8:**
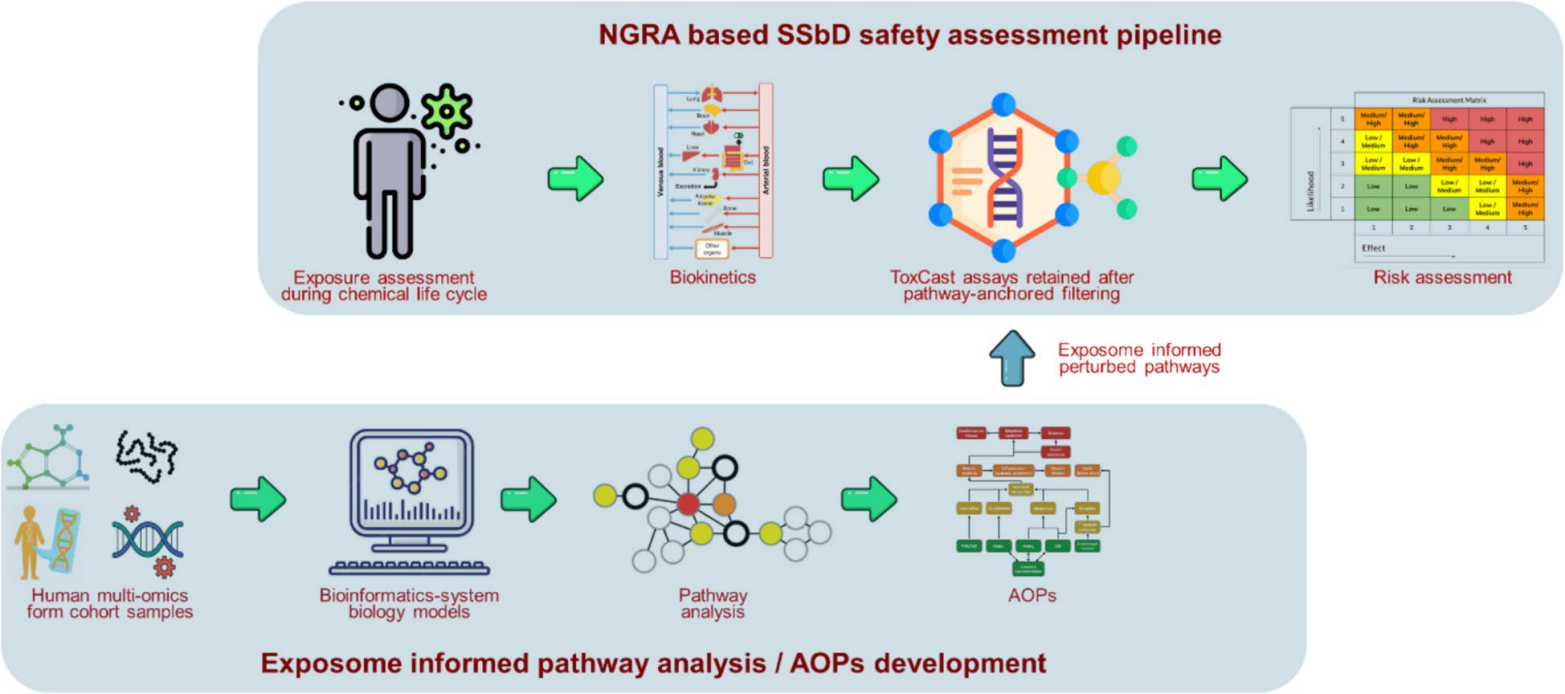
Exposome-informed NGRA pipeline for SSbD safety assessment. Schematic overview of an exposome-informed, next-generation risk assessment (NGRA) pipeline supporting Safe and Sustainable by Design (SSbD) decisions. Upper panel: exposure assessment across the chemical life cycle is translated into internal dose metrics via biokinetics/PBPK modelling, which are compared against pathway-anchored high-throughput screening (HTS) bioactivity data (e.g. ToxCast) to derive risk-based stage-gate decisions. Lower panel: human cohort exposome data, including multi-omics profiles, are analysed using bioinformatics and systems biology to identify perturbed biological pathways and inform AOP development. These exposome-derived pathway insights feed back into the NGRA pipeline, guiding the selection and interpretation of relevant HTS/NAM endpoints and strengthening the mechanistic anchoring of SSbD safety assessments

From a policy perspective, these findings directly support the objectives of the EU Chemicals Strategy for Sustainability, which calls for moving safety upstream, avoiding regrettable substitutions, and embedding human-relevant evidence into innovation decisions. The PBPK–HTS–AOP integration demonstrated here exemplifies how the PARC SSbD toolbox can operationalise these goals in practice, enabling early rejection of structurally similar but biologically problematic alternatives such as BPAP, while prioritising candidates like isosorbide that decouple function from engagement of vulnerable biological pathways. More broadly, this approach illustrates how SSbD can evolve from a qualitative design principle into a quantitative, prevention-oriented decision framework. In the present case study, integrating exposome analysis into the SSbD framework reinforces mechanistic relevance by anchoring high-throughput screening outputs to real-world human biology via AOPs [88], thereby aligning early hazard evaluation with human disease pathways. Exposome-derived multi-omics signatures from human cohorts provide dense, pathway-level fingerprints of environmentally induced perturbations, highlighting which molecular initiating events (MIEs) and AOPs are most pertinent to human health outcomes. These pathway perturbation signatures, gleaned from human omics data, effectively bridge observational exposure science with mechanistic toxicology, pinpointing the key biological targets that should be prioritized when selecting and interpreting NAM data [89]. Anchoring new approach methodologies (NAMs) to these human-relevant pathways ensures that in vitro bioactivity findings are tied to meaningful adverse outcome trajectories [51, 90], thereby improving the predictive power and human relevance of early hazard screening. For example, if exposomic studies underscore estrogen receptor–mediated pathways as drivers of bisphenol A’s effects, then AOPs involving this MIE become central in evaluating safer alternatives like BPAP or isosorbide. By leveraging such exposome-guided mechanistic anchors, SSbD practitioners can more confidently differentiate truly benign design options from “regrettable” substitutes that still trigger critical toxicity pathways, thereby focusing innovation on inherently safer chemistry. To translate these mechanistic insights into actionable safety margins, internal dosimetry refinement is needed, where physiologically based pharmacokinetic modeling or biomonitoring can map NAM-derived effect thresholds onto realistic tissue exposure levels, effectively connecting NAM potency to real-world risk. While this step grounds hazard signals in actual exposure contexts, the primary advance remains mechanistic. By focusing SSbD decision-making on the biological mechanisms most relevant to human health, exposome-informed pathway anchoring substantially enhances the scientific rigor and preventive potency of early-stage safety evaluations [88], ensuring that design-phase choices are guided by mechanisms known to drive actual adverse health outcomes in human populations. Integrating exposome analysis into the SSbD framework anchors high-throughput screening outputs to real-world human biology via AOPs [88], aligning early hazard evaluation with human disease pathways. Exposome-derived multi-omics signatures identify which molecular initiating events and AOPs are most pertinent to human health, bridging observational exposure science with mechanistic toxicology [89]. Anchoring NAMs to these human-relevant pathways ties in vitro bioactivity findings to meaningful adverse outcome trajectories [51, 90]. For instance, estrogen receptor–mediated AOPs become central when evaluating BPA alternatives such as BPAP or isosorbide. PBPK modelling maps NAM-derived thresholds onto realistic tissue exposures, ensuring design-phase decisions are guided by mechanisms known to drive adverse health outcomes [88] (Fig. 8).

## Policy and regulatory implications

Traditional regulatory regimes have typically evaluated chemicals one by one [2]. This does not fit well the life-course exposome perspective, which shows that humans are continuously exposed to complex mixtures of environmental stressors, concurrently or sequentially, often with health effects that go beyond additivity (Rappaport and Smith [19]. SSbD responds by shifting from managing single substances to proactively eliminating hazards and curbing exposure at source. At the same time, incentives for green chemistry, such as tax breaks, grants or streamlined approvals, can steer industry toward safer alternatives, helping bridge the gap between experimental toxicology and real-world sustainable materials development [2]. Mixture-oriented and exposome-informed regulation, supported by biomonitoring, targeted toxicology and life course exposure data, can further align bans and restrictions with actual population exposure patterns [7].

The life course exposome framework also underlines that susceptibility is not uniform across time: prenatal development, early childhood and other critical windows are especially vulnerable to even low-level neurotoxic or endocrine-disrupting exposures, with effects that may manifest much later [6]. Future regulations are therefore likely to adopt more protective limits for pregnant women, infants and other high-risk groups, for example by incorporating vulnerability explicitly into reference dose derivation [6]. Occupational settings require similar attention: many workers experience repeated exposures far above those of the general population, and embedding biomonitoring into occupational standards can provide a more realistic picture of individual risk and trigger earlier intervention [91]. At the community level, integrating exposure data into local assessments helps identify neighborhoods disproportionately affected by emissions from industrial facilities or waste sites, supporting tighter emission controls or, in extreme cases, relocation of hazardous activities [21]. In this way, tracking exposures over the life course helps direct regulatory protection to those most at risk.

Enabling SSbD in practice depends on a robust information infrastructure linking hazard, exposure and life-cycle data. Stronger transparency rules can enhance both scientific understanding and public awareness of how chemicals are used and what risks they pose [92]. Full ingredient disclosure in consumer products would allow researchers and authorities to track which substances occur in everyday items and to prioritize problematic chemicals for early action. Public access to life-cycle data on resource use, energy demand, emissions and waste would similarly support independent evaluation of the environmental and health implications of new chemicals or technologies, and allow civil society to hold producers accountable. Open repositories for biomonitoring data offer another pillar: they equip scientists and communities with evidence on internal chemical burdens, enable the detection of hotspots near industrial sources, and can link rising body burdens to local emission patterns [93, 94]. Taken together ingredient transparency, open LCAs and accessible biomonitoring, these measures create a knowledge base in which exposomic evidence directly informs SSbD decisions, aligning commercial innovation with public health protection and ecological sustainability.

## Future directions and research priorities

SSbD is emerging as an approach in chemical design and product development that weaves safety for people and the environment into the earliest stages of innovation [15]. Beyond assessing legacy substances, it opens space for creating new chemistries that are inherently less hazardous and more sustainable. To do this credibly, SSbD has to rest on a convergent toxicological evidence base that links structure, exposure and biological response across the life course. Exposomics, NAMs and life-cycle information together provide that foundation, while decision-support tools such as MCDA can help organise the evidence and make trade-offs transparent [95, 96].

Overall, the SSbD approach encourages an iterative (re)design process in which new findings from NAMs and exposome research continually refine both the evaluation of existing substances and the design of new ones. When emerging evidence shows that a particular chemical class disproportionately affects specific life stages or target organs, SSbD practitioners can use this mechanistic insight to down-select candidates early, avoiding regrettable substitutions. In parallel, if exposome or biomonitoring data reveal unexpectedly high workplace or community exposures, decision-support frameworks such as MCDA can flag these materials as priority targets for redesign, restriction or substitution [15]. In this way, toxicological convergence, rather than any single tool, becomes the core engine driving Safe and Sustainable by Design.

## Conclusion

The promise of SSbD joined with the life-course exposome rests on execution: a repeatable workflow that starts from use-driven exposure, designs away mechanistic risk using NAMs and AOPs, verifies predictions with physiology-based kinetic (PBK) modeling and biomonitoring, and iterates when reality diverges [60]. Applied consistently, this advances the Chemicals Strategy for Sustainability by avoiding rather than shifting problems. SSbD becomes less a label than a discipline: design, measure, learn and redesign until function, health protection and sustainability co-exist in the same molecule and product system. The life-course exposome reveals when, where and in whom substances have the most adverse effects, highlighting critical windows and over-burdened communities. SSbD provides the stage-gated, life-cycle framework that can translate those insights into upstream design constraints, substitution choices and product stewardship. Together these approaches offer a practical route to reducing disease burdens linked to chemical exposures and promoting environmental fairness by targeting the worst hazards and exposure routes. Exposome data help SSbD practitioners prioritise chemicals, use patterns and life-cycle stages, and test whether “safe-by-design” claims hold in real life, while SSbD gives exposome researchers a lever to translate biomonitoring and mixture evidence into prevention at the source. Achieving this synergy is not automatic: it depends on cross-disciplinary collaboration, continued method development and policies that reward hazard avoidance and exposure reduction over reactive risk management. Materials scientists, chemical engineers and toxicologists need timely data on how new substances affect pregnant women, infants, workers and communities, while regulators need clear criteria that embed NAMs, AOPs and exposomic indicators into decision-making. Overall, combining the life-cycle view of SSbD with the life-course lens of exposome science offers a robust roadmap under the EU Chemicals Strategy for Sustainability and the Green Deal: eliminate hazards early, track real exposures over time and adjust design so that healthier populations and cleaner environments become the default outcome of innovation.

## Data Availability

All datasets analysed during the current study are publicly available or derived from published sources. High-throughput screening (HTS) bioactivity data were retrieved from the US EPA CompTox Chemicals Dashboard (https://comptox.epa.gov), and assay selection and curation procedures are described in the Methods section and summarised in Table 2. Physicochemical and toxicokinetic parameters used for PBPK modelling were obtained from peer-reviewed literature cited in the manuscript. Exposure modelling was conducted using the INTEGRA platform, with model structure, input assumptions, and scenario definitions described in the Methods section. Life-cycle assessment inputs were derived from published databases and referenced literature. To ensure reproducibility, the curated assay lists, PBPK parameter sets, model input files, and summary outputs supporting the main findings will be deposited in a publicly accessible repository (e.g. Zenodo) upon acceptance of the manuscript. The repository DOI will be provided in the final published version. No new human subject data were generated in this study.

## Abbreviations

AOP: Adverse outcome pathway
AOPs: Adverse outcome pathways
AC50: Half maximal activity concentration
ADME: Absorption, distribution, metabolism and excretion
BPA: Bisphenol A
BPAP: Bisphenol AP
BPS: Bisphenol S
Css: Steady-state plasma concentration
Cmax: Maximum plasma concentration
EF: Environmental footprint
ER: Estrogen receptor
ERα: Estrogen receptor alpha
ERβ: Estrogen receptor beta
ERC: Environmental release category
EU: European Union
EWAS: Environment-wide association study
FCM: Food contact material
GWP: Global warming potential
HTS: High-throughput screening
INTEGRA: Integrated Exposure Model Platform
LCA: Life-cycle assessment
MCDA: Multi-criteria decision analysis
MIE: Molecular initiating event
NAM: New approach methodology
NAMs: New approach methodologies
NGRA: Next-generation risk assessment
NTA: Non-targeted analysis
PBK: Physiologically based kinetics
PBPK: Physiologically based pharmacokinetic
PC: Polycarbonate
PEC: Predicted environmental concentration
PROC: Process category
qIVIVE: Quantitative in vitro to in vivo extrapolation
QSAR: Quantitative structure–activity relationship
RCR: Risk characterization ratio
SSbD: Safe and sustainable by design
TSHR: Thyroid-stimulating hormone receptor
US EPA: United States Environmental Protection Agency
